# Entrance Examinations for Medical Colleges

**Published:** 1886-09

**Authors:** 


### *ENTRANCE EXAMINATIONS FOR MEDICAL COLLEGES.*

The opening of the annual session of medical colleges is near at hand. Annual announcements have been freely distributed, offering liberal inducements to all who apply. Medical students scan the claims thus presented, naturally, and often from necessity, inclining to economy of expense and of time, and also giving heed to the facility with which the medical degree can be obtained. The commercial spirit of the age controls aspirants for medical honors, as in other departments of life. If colleges make bids for numbers, their terms of admission and curriculum of study are trimmed to catch the breeze. As a consequence, the medical colleges of this country are filled with all sorts of material, and the ranks of the profession receive an annual influx of half-educated and imperfectly prepared men, the majority of whom, fortunately for the public, find their mistake in the choice of a vocation, only after testing their qualifications in the crucible of practice and experience.

Indications of a reaction from this state of things, the fruition of which has been a disgrace to the American profession, and demonstrates the faults of our system of medical education, have been cropping out for several years, assuming a definite shape in the higher curriculum of study, and in the entrance examinations of several of our leading medical schools.

Harvard and Yale have placed an excellent example before the profession in establishing an entrance examination, and also a graded course of study. And the conditions of admission are not, in our opinion, any too severe. Harvard requires the student to "pass a satisfactory examination in English, Latin, physics, and some one of the electives, Botany, French, German, elementary algebra or plane geometry." Yale requires "algebra to quadratics, Euclid, two books, the metric system and elementary physics." Niagara University, the medical department of which is located in this city, requires an "examination in the branches of a good English education, including mathematics, English composition and elementary physics, or natural philosophy and in Latin, including 'Arnold's First Latin Book,' as its equivalent." This growing institution has a graded course of study, patterned after that of its sister universities.

It is not necessary to enter upon the reasons for these steps for higher medical education. Will the profession support the movement? Heretofore the responsibility of determining the intellectual qualifications of students in medicine has devolved upon individual members of the profession. The result is apparent. If the present state of medical education is primarily due to indifference or carelessness upon the part of individual physicians, and if medical colleges have prospered by catering to such a lamentable want of foresight, then it may be inferred that any movement, which has for its ulterior object the increase of professional culture, must depend for its support upon the educated and cultured members of the profession. And here we may expect that selfish interests will have their influnce. The discreditable spectacle, observed in this city within a few years, of an organized effort within the profession, instigated and backed by the faculty of a medical college, in which higher education is considered a chimera for visionary enthusiasts, against the establishment of a medical college, pledged to higher medical education, demonstrates plainly that sympathy and support cannot be expected from a large and influential portion of the profession.

We have an abiding faith in the ultimate success of these principles. The initial steps have already been taken by several of the best colleges. The fact that an entrance examination, a graded course of study, and a rigid examination for the degree, by a board, independent of the teaching body, are adopted by several schools, has a significant meaning. The advance needs to be backed up by a strong professional sentiment, which has already taken root and is growing with remarkable rapidity in this country. If, against mercenary and selfish influences, gratifying progress has been made within the last decade, we may safely anticipate that the general recognition of the necessity of superior intellectual culture for the matriculant in medicine will be established 'ere another decade has passed away.

